# Research and testing of a robot vision-based perception method for assessing corn sowing quality

**DOI:** 10.3389/fpls.2026.1804475

**Published:** 2026-04-29

**Authors:** Wei Zeng, Hao Wang, Yuejin Xiao, Bingxin Yan, Zhijun Meng, Chengtao Zhang

**Affiliations:** 1 School of Mechanical and Automotive Engineering, Guangxi University of Science and Technology, Liuzhou, China; 2 Research Center of Intelligent Equipment, Beijing Academy of Agriculture and Forestry Sciences, Beijing, China; 3 Nongxin Technology (Beijing) Co., Ld., Beijing, China

**Keywords:** 3D machine vision, keypoint detection, seedling-stage plant spacing, sowing quality, YOLOv11-pose

## Abstract

To address the low efficiency of manual inspection for corn sowing quality, which is labor-intensive and time-consuming, this study proposes an automatic seedling-stage plant-spacing measurement method based on three-dimensional machine vision. A high-clearance mobile platform equipped with a ZED 2i stereo camera and an industrial computer was developed to acquire RGB images and depth information of corn seedlings in the field in real time. Using the YOLOv11-Pose model, plant keypoints were detected and localized; combined with camera calibration and 3D reconstruction techniques, inter-plant distances were computed automatically. A sowing-quality evaluation framework was then established to enable automated analysis of the Quality of Feed Index (QFI), Multiple Index (MUL), Miss Index (MI), and coefficient of variation. Experimental results indicate that the system operates effectively under three preset plant spacings (15 cm, 20 cm, and 25 cm), achieving a keypoint-detection mAP@0.5 of 0.990 and an mAP@0.5:0.95 of 0.989. In sowing-quality evaluation, the qualified indices produced by the system were 77.83%, 80.36%, and 82.46%, respectively, and the Quality of Feed Index (QFI), Multiple Index (MUL), Miss Index (MI), and coefficient of variation showed trends consistent with manual measurements. The proposed method enables efficient, nondestructive detection of seedling-stage plant spacing and sowing quality, providing reliable technical support for precision sowing and field management.

## 1 Introduction

Accurate detection and evaluation of corn sowing quality is a critical step for ensuring uniform field emergence and achieving high and stable corn yields. The spatial configuration of row spacing and plant spacing alters canopy structure and the distribution of light energy, thereby affecting photosynthetic productivity and yield formation (Ding et al., 2025). Measurements of actual plant spacing at the seedling stage can provide direct evidence for field operations such as thinning and replanting, and can also offer objective, quantitative data to support evaluations of planter operating performance and optimization of sowing parameters (Ren et al., 2024). Therefore, developing a rapid, accurate, and nondestructive automated method for measuring seedling-stage corn plant spacing is of substantial importance for improving sowing operation quality and advancing precision management in corn production.

At present, inter-seedling spacing is still obtained primarily through manual field measurements, or by manually “digging seeds” after sowing to determine seed drop-point information. Such approaches are inefficient and labor-intensive, and they can readily disturb the soil cover structure and affect subsequent emergence, making them difficult to scale for large-area, precision-oriented agricultural management (Xie et al., 2025). From both industrial and academic perspectives, sowing-quality inspection technologies have evolved from single-sensor approaches (e.g., photoelectric/capacitive sensing) toward multi-sensor fusion and online monitoring driven by machine vision and artificial intelligence. For example, multi-sensor data fusion can improve the stability and accuracy of monitoring sowing depth under high-speed precision planting (Wang et al., 2025b), and variable-speed/variable-rate electronic seed-metering mechanisms, along with mis-sowing detection and re-sowing strategies, have also been explored in recent studies (Elwakeel et al., 2025). However, most existing systems evaluate sowing quality using seed passage states or seed drop points “at the moment of sowing,” implicitly assuming that the seed drop point is equivalent to the emergence location. As a result, they insufficiently account for factors such as in-field seed displacement, soil-cover disturbance, and variability in emergence rate (Nardon and Botta, 2022; Xie et al., 2025).

For monitoring during the sowing operation, extensive research in recent years has focused on seed spacing, seed-metering performance, and sowing-depth monitoring under high-speed conditions. (Gao et al., 2024) proposed a corn seed-spacing detection method based on image stitching and YOLOX, in which a speed–frame-rate linkage is used to generate a panoramic seedbed image and enable seed recognition and localization. (Zhao et al., 2025) combined an improved YOLOv5s with SiamRPN++ to track seed trajectories inside the seed-metering chamber and evaluate operational performance. For online measurement of plant-spacing parameters, (Xie et al., 2025), proposed a wireless remote real-time ranging system by fusing speed sensing and seed sensing, providing a new pathway for online sowing-quality assessment. Although these methods achieve strong monitoring performance at the seed level, they still struggle to directly bridge the discrepancy between “seed drop point” and “emergence location.” Consequently, their ability to faithfully represent seedling-stage population uniformity remains limited, as they inherently fail to account for post-planting dynamic errors induced by complex terrain variations and delayed emergence (Vong et al., 2022; Ren et al., 2024).

In recent years, advances in computer vision and deep learning have made it increasingly feasible to directly identify corn seedlings, diagnose missing plants, and conduct spatial analyzes at the seedling stage. (Ren et al., 2024) combined an improved YOLOv8 model with Voronoi analysis on UAV imagery to evaluate seedling status quality. (Gao et al., 2025) proposed Corn YOLOv8n for detecting missing plants in UAV imagery, and (Yang et al., 2025) further integrated semi-supervised learning with geolocation to enable missing-plant monitoring and coordinate localization. To address complex near-ground backgrounds and weed interference, Wang et al. (2025) proposed a vision- and AI-based automatic thinning method for corn seedlings, achieving high-precision localization of plant centers via zero-sample annotation and an improved keypoint network (PCK = 97.66%). (Wu et al., 2025) performed seedling-stage classification based on H-RT-DETR; Feng et al. (2025b) designed a lightweight, field-deployable YOLOv8n for real-time seedling detection; (Chu et al., 2025) enhanced seedling-stage precise localization using CornStar YOLO; and (Wang et al., 2025a) achieved seedling detection and counting in complex field environments. Nevertheless, these existing references predominantly rely on two-dimensional measurements, which present significant limitations for precise spatial quantification under realistic field conditions. During actual agricultural operations, continuous mechanical vibrations and complex terrain variations dynamically alter the camera perspective, introducing severe scaling errors and geometric distortions in 2D imagery. Furthermore, the varying morphological postures and irregular leaf extensions of developing corn seedlings cause the geometric centers of traditional 2D bounding boxes to fluctuate significantly, making them unreliable for precise plant-to-plant distance calculations. Because 2D vision fundamentally lacks depth perception, it cannot correct these spatial artifacts. Therefore, incorporating 3D vision is strictly necessary to acquire absolute physical coordinates, mitigate vibration and posture-induced localization errors, and achieve accurate seedling-stage spatial measurements. To improve the robustness of within-row spatial-structure extraction, progress has also been made in crop-row detection and spatial-constraint modeling, such as the row–column attention end-to-end crop-row detection framework by (Li et al., 2024), the ALNet corn-row detection network by (Feng et al., 2025a), and row-structure extraction and spatial modeling approaches that fuse depth/3D information (Gong and Zhuang, 2024; Zhou et al., 2024; Afzaal et al., 2025; Ban et al., 2025). These efforts provide important methodological foundations for 3D localization and plant-spacing measurement at the seedling stage; however, automated 3D plant-spacing detection and a quantitative evaluation system for sowing quality under complex field conditions still require further refinement.

To address the spatial discrepancy inherent in seed-drop monitoring and the dynamic measurement errors of two-dimensional vision, this study proposes an automated, non-contact method for measuring seedling-stage corn plant spacing based on three-dimensional machine vision. By employing an advanced object detection model to localize plant centers and synchronously fusing RGB imagery with real-time depth data, this method projects two-dimensional targets into a unified three-dimensional physical coordinate system, thereby mitigating systematic measurement errors. Furthermore, a sowing-quality evaluation framework is established to enable automated analysis of the Quality of Feed Index (QFI), Multiple Index (MUL), Miss Index (MI), and coefficient of variation directly from the three-dimensional spatial data. Ultimately, this approach advances the assessment paradigm from monitoring the mechanical sowing process to quantifying the established seedling population, providing reliable technical support for precision sowing and field management.

## 2 Materials and methods

### 2.1 Overall design and implementation of the detection system

#### 2.1.1 Overall design of the sowing scheme

**Figure 1:**
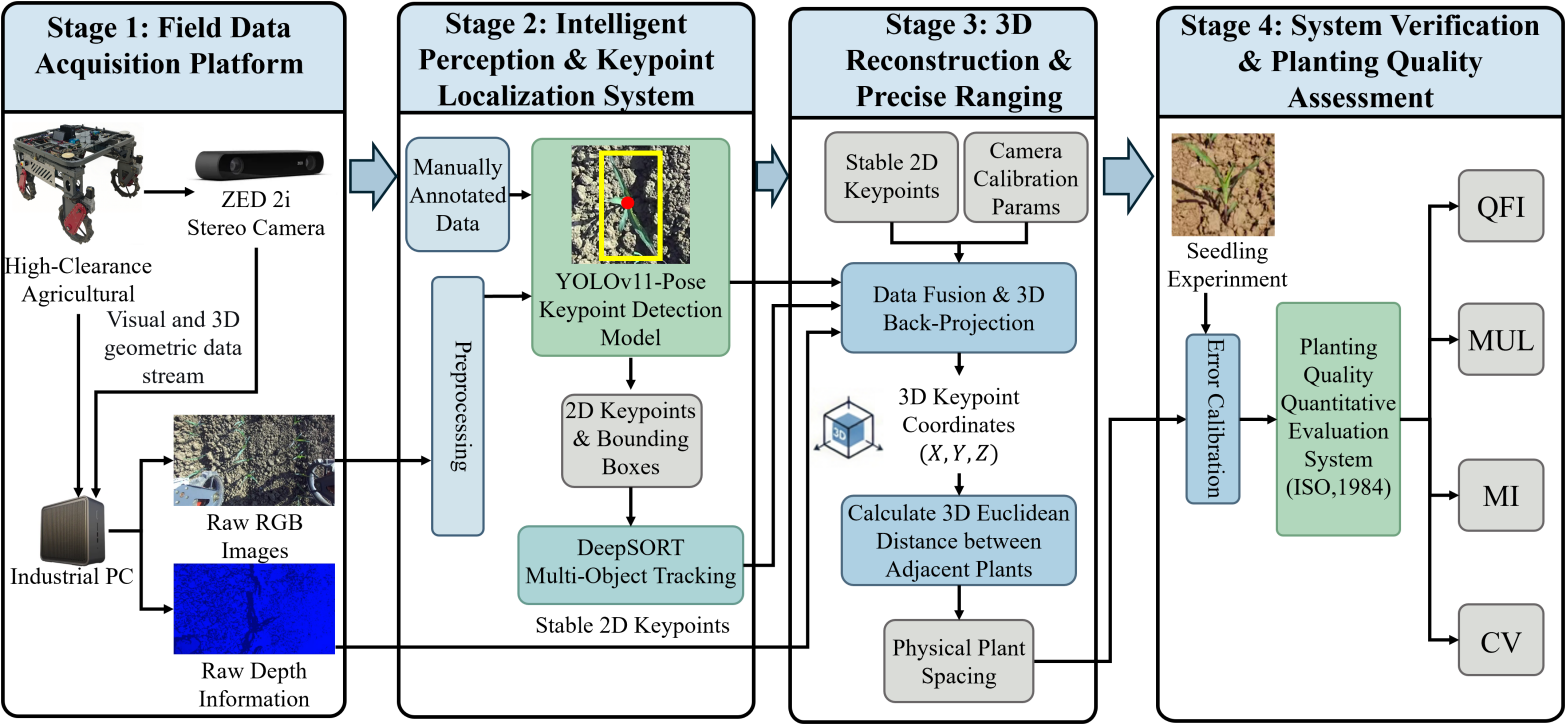
Flowchart overview of the proposed method.

To enable automated and accurate detection of corn seedling plant spacing, this study developed a complete 3D machine vision–based detection system, as illustrated in **Figure 1**. The system is centered on a ZED 2i stereo camera mounted on a high-clearance robot. By acquiring top-view RGB images and depth information of corn seedlings synchronously along crop rows and applying standardized preprocessing, the system provides high-quality data for subsequent analysis.

The core of the study lies in constructing a keypoint-detection model and accurately computing three-dimensional plant spacing. First, the centroid of each corn seedling was annotated as the keypoint, and a YOLOv11-Pose model was trained and deployed to achieve automated plant recognition and precise localization in field conditions. Building on these outputs, the system tightly integrates camera calibration parameters with depth information to back-project the two-dimensional keypoints into three-dimensional space. While mounting a camera vertically at a fixed height (e.g., 1.15 m) might suggest that a simpler 2D pixel-distance calculation with a fixed scale factor is feasible, this approach is fundamentally limited in dynamic field environments. In practice, microtopography variations (such as soil ridges and clods), differences in individual plant heights, and the pitch and roll of the moving high-clearance platform constantly alter the absolute camera-to-target distance. Since the vision sensor relies on perspective projection, these Z-axis fluctuations cause continuous scaling variations across the 2D image plane. By fusing 2D object detection centers with real-time depth data, the proposed 3D methodology projects targets into a unified physical coordinate system, effectively eliminating the systematic scaling errors inherent in fixed-parameter 2D measurements.

To verify the reliability and practicality of the system, measurement errors were calibrated using artificial-seedling experiments. Based on the resulting plant-spacing estimates, the system automatically computes the QFI, MUL, MI, and coefficient of variation, thereby enabling a quantitative evaluation of sowing quality. Overall, the proposed approach establishes an automated closed loop spanning field data acquisition, plant localization, 3D plant-spacing computation, and sowing-quality assessment, providing a robust technical solution for precise monitoring of crop establishment quality.

In summary, this study integrates 3D vision, deep learning, and automated analytics to develop a full-process, noncontact, and automated detection framework that encompasses field data acquisition, seedling identification, 3D distance measurement, and intelligent evaluation of sowing quality.

#### 2.1.2 Field data acquisition system for a high-clearance weeding robot

**Figure 2:**
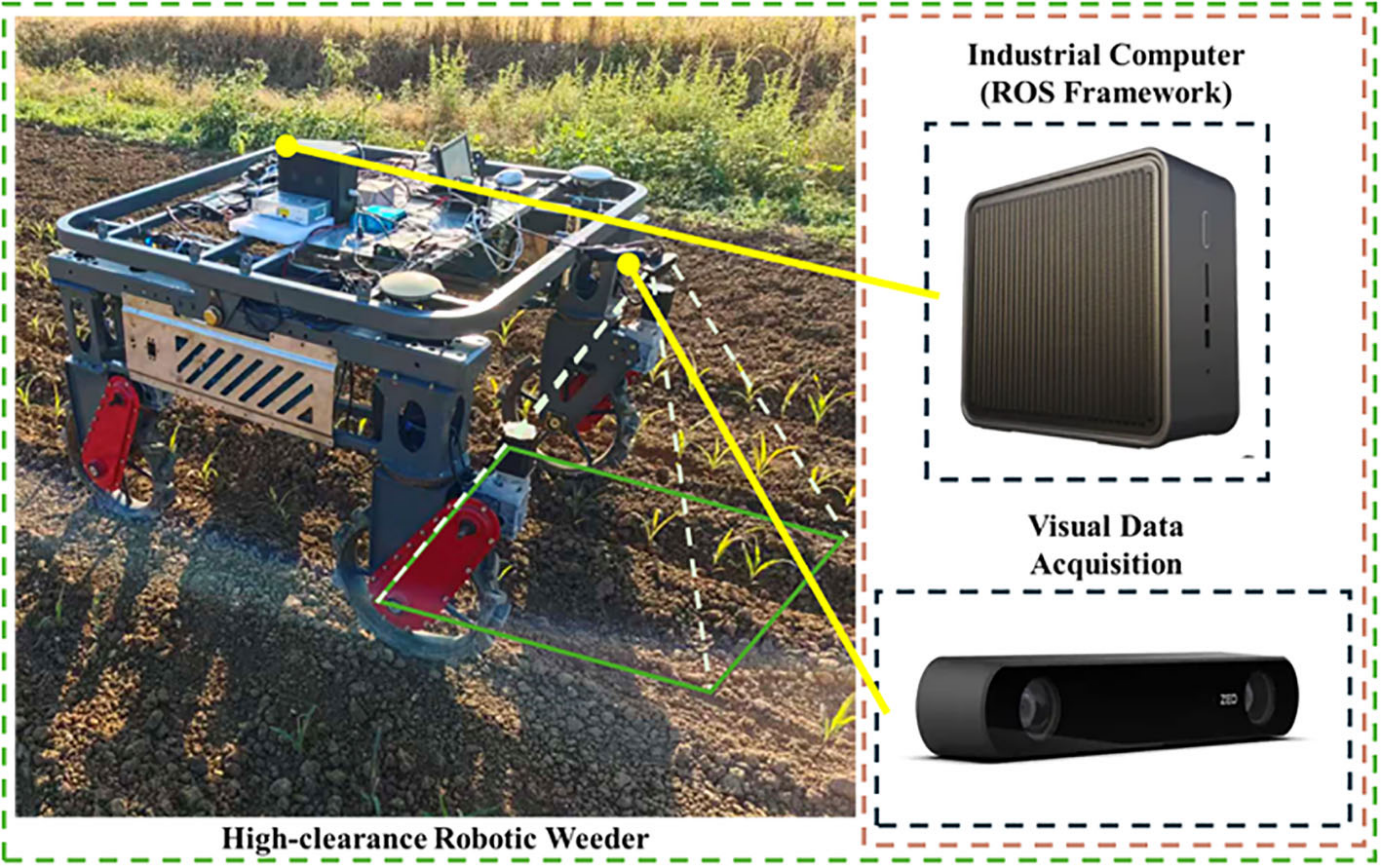
Field data acquisition system for the high-clearance weeding robot.

This study focuses on high-resolution top-view images of corn seedlings and investigates an intelligent, deep-learning-based approach for plant keypoint detection. As shown in **Figure 2**, a stable and controllable field data-acquisition system was established to ensure image-data quality and the accuracy of subsequent analyzes. The system utilizes a ZED 2i stereo camera as the primary vision sensor, mounted on a high-clearance weeding robot platform, and is centrally controlled and processed in real time by an Intel NUC 9 Pro industrial computer equipped with a dedicated NVIDIA RTX 3060 GPU. To ensure robust real-time performance during field deployment, the trained object detection model was exported and accelerated using the TensorRT inference engine.

The ZED camera was mounted at the center of the robot’s front crossbeam, with the lens oriented vertically downward at a nominal fixed height of 1.15 m above the ground; however, the actual camera-to-target distance fluctuated dynamically due to field terrain variations and machine posture. According to the manufacturer’s specifications and our preliminary calibration, the expected depth measurement error of the ZED 2i camera at this absolute distance (1.15 m) is approximately 1.0% to 1.5% (i.e., ~11.5 to 17.2 mm). This centimeter-level accuracy remains well within the acceptable tolerance for reconstructing the spatial distribution of field plants at the macro level. Images were acquired at a resolution of 1100 × 620 pixels with a frame rate of 15 FPS, balancing fine visual detail with real-time processing capability. The industrial computer synchronously controls the acquisition and storage of RGB images and depth data, and integrates real-time preprocessing and keypoint-detection algorithms, enabling end-to-end automation from data capture to preliminary analysis.

The acquisition system was designed to ensure adaptability to field conditions, data consistency, and experimental repeatability, thereby providing a reliable data foundation for subsequent corn-seedling keypoint detection and plant-spacing computation.

### 2.2 Vision-based detection method for corn seedlings

#### 2.2.1 Data acquisition

**Figure 3:**
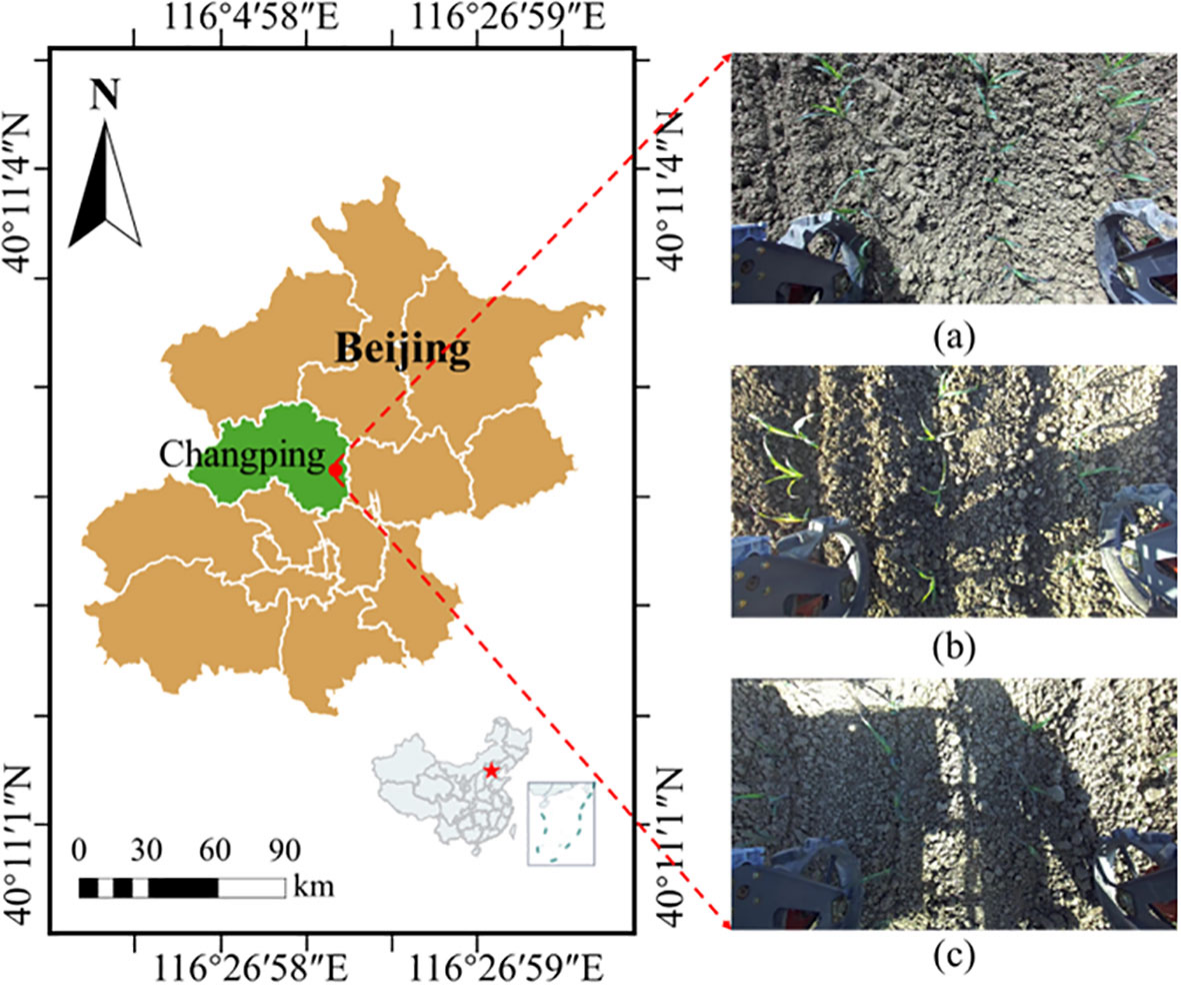
Data acquisition. **(a)** under front-lighting conditions; **(b)** under side-lighting conditions; **(c)** under shaded conditions.

The image data used for corn seedling detection and 3D plant spacing measurement in this study were collected in October 2025, as shown in **Figure 3**, at the experimental fields of the National Precision Agriculture Demonstration Base in Beijing, China (40°11′2″N, 116°26′59″E). The test crop was the corn cultivar NK815, sown under conventional management using a four-row vacuum planter manufactured by Beijing Debangte Agricultural Machinery Co., Ltd. Data acquisition covered multiple lighting conditions, including shadows, side lighting, and front lighting, which are representative of typical field illumination scenarios. In total, 2,500 top-view images of corn seedlings at the five-leaf stage were collected at a resolution of 1100 × 620 pixels to ensure model generalization under complex lighting. Images were captured using a stereo ZED camera mounted on a high-clearance weeding robot to record realistic top-down field scenes of corn seedlings. As illustrated in **Figure 1**, the resulting dataset spans shadowed, side-lit, and front-lit conditions, providing a comprehensive and representative foundation for the corn-seedling keypoint detection task.

#### 2.2.2 Annotation of corn seedling centroids

In this study, Labelme was used to perform instance-level annotation of corn seedlings in field images. The annotations included an object bounding box and a single keypoint. Bounding boxes were labeled as rectangles, defined as the minimum enclosing rectangle of the plant’s visible regions in the image, including leaves, the leaf sheath, and the stalk. The keypoint represents the location of the plant’s apical growing point in the image, and only one keypoint was annotated within each bounding box.

**Figure 4:**
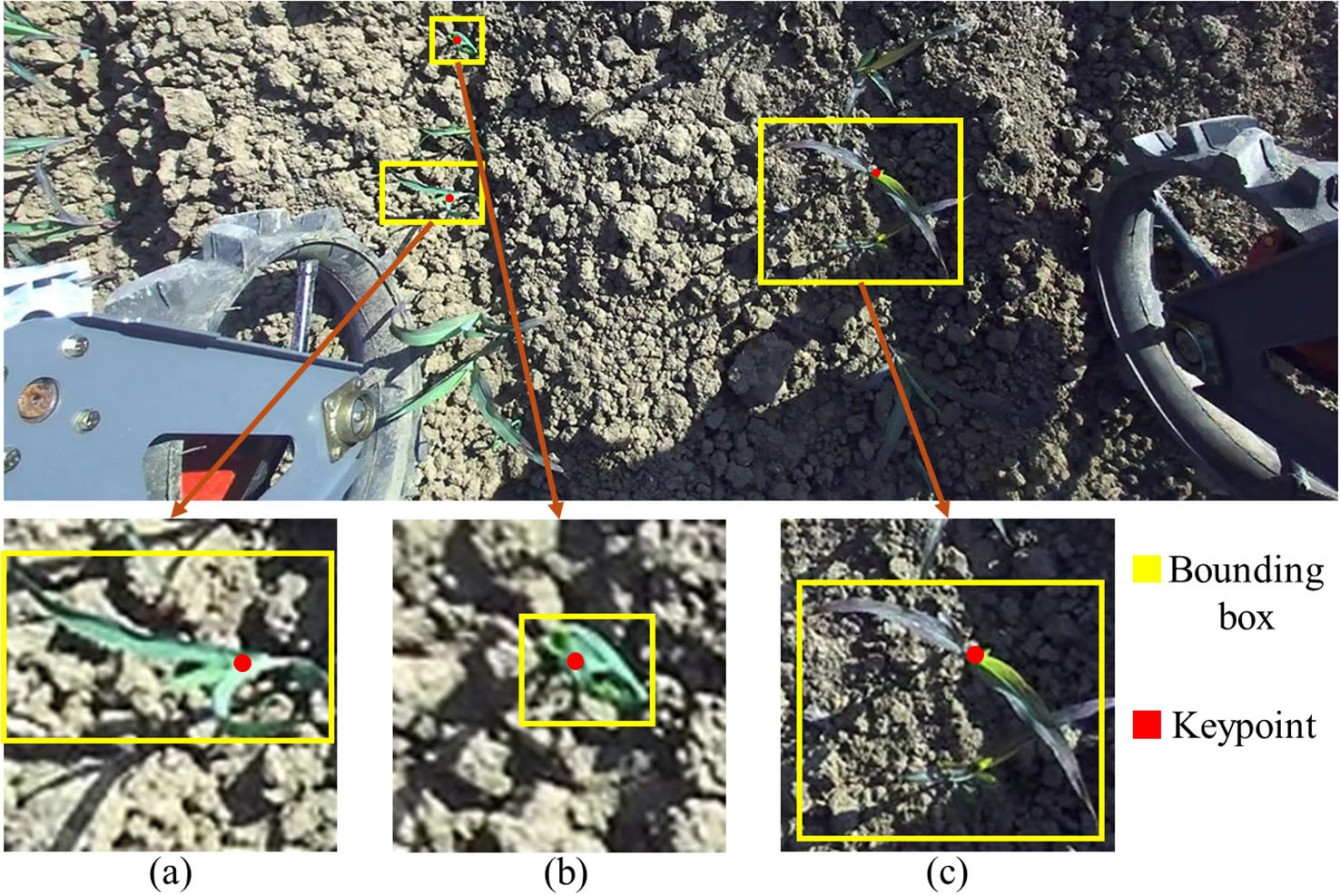
Centroid annotation of corn seedlings under different poses. **(a)** Medium-sized corn seedlings, **(b)** Small-sized corn seedlings, and **(c)** Large-sized corn seedlings.

Because corn seedlings in the field exhibit pose variations and occlusions (as shown in **Figure 4**), keypoint annotation followed a hierarchical set of rules: (1) when the central whorl (the core region of the heart leaves) is clearly visible, as illustrated in **Figure 4c**, the keypoint is placed at the geometric center of the whorl core; (2) when the whorl core is not visible, as illustrated in **Figure 4a**, to ensure consistency and practicality, a stable alternative landmark is selected near the plant’s main axis—specifically, the junction between the leaf sheath and the stalk at a visible leaf attachment point; and (3) when seedlings are small and the core is difficult to observe, as illustrated in **Figure 4b** , the keypoint is placed at the center of the seedling.

The annotation results were saved in JSON format and, prior to model training, converted to the TXT label format required by YOLOv11-Pose. Coordinates were represented either in pixel units or as values normalized to the image dimensions. The dataset was then split into training, validation, and test sets at a ratio of 8:1:1.

#### 2.2.3 Keypoint detection model for corn seedlings

This study employs the YOLOv11-Pose model to perform pose estimation on corn seedling images. As the official pose-estimation variant of YOLOv11, this model retains the YOLO family’s single-stage, high-efficiency detection framework while extending it with keypoint regression capabilities, making it well suited for multi-object pose analysis in complex scenes. Its objective is to achieve automated and quantitative characterization of corn seedling growth posture by identifying and localizing structured keypoints of the plant.

**Figure 5:**
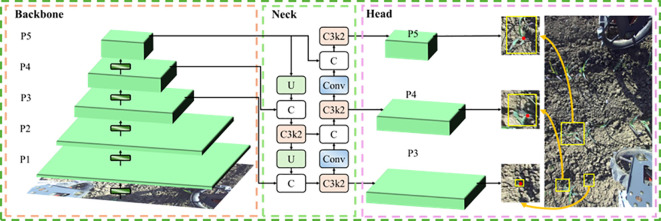
YOLOv11-pose network architecture.

As shown in **Figure 5**, YOLOv11-Pose follows the canonical YOLO architecture and consists of three main components: a backbone network (Backbone), a feature fusion network (Neck), and a prediction head (Head). The backbone is a deep convolutional neural network responsible for extracting hierarchical features from the input image. The feature fusion module adopts a multi-scale pyramid structure that integrates semantic information across different levels to construct feature maps suitable for multi-scale object detection. The prediction head uses a decoupled design with parallel detection and pose branches. The detection branch typically includes multiple detection heads corresponding to large-, medium-, and small-scale receptive fields: the head for large corn seedlings is connected to deeper feature maps and leverages their rich semantics for recognition; the head for medium seedlings connects to intermediate feature maps to balance semantic content and spatial detail; and the head for small seedlings connects to shallow, high-resolution feature maps to capture fine-grained seedling targets. The detection branch ultimately outputs bounding boxes and class confidences. In coordination with the detection branch, the pose branch regresses the keypoint coordinates for each detected instance. This architecture supports end-to-end inference, enabling simultaneous and accurate multi-scale plant detection and keypoint localization in complex seedbed images.

#### 2.2.4 Multi-object tracking methods

Deep SORT (Wojke et al., 2017) is an enhanced algorithm developed from the classical SORT (Bewley et al., 2016) framework, with its primary contribution being the introduction of an appearance-feature matching module based on deep convolutional neural networks. By extracting and comparing deep appearance descriptors of targets across frames, this module substantially improves the robustness of cross-frame identity association, markedly reduces the frequency of identity switches (ID switches), and strengthens the ability to preserve identity consistency under challenging conditions such as illumination variation and partial occlusion. This capability is particularly important for continuous tracking of corn seedlings because, during field operations, a moving high-clearance weeding robot may induce dynamic occlusions or shadow artifacts over seedlings. Through continuous re-identification of target appearance, the system can reliably lock onto specific corn seedlings, thereby preventing false associations and tracking loss.

**Figure 6:**
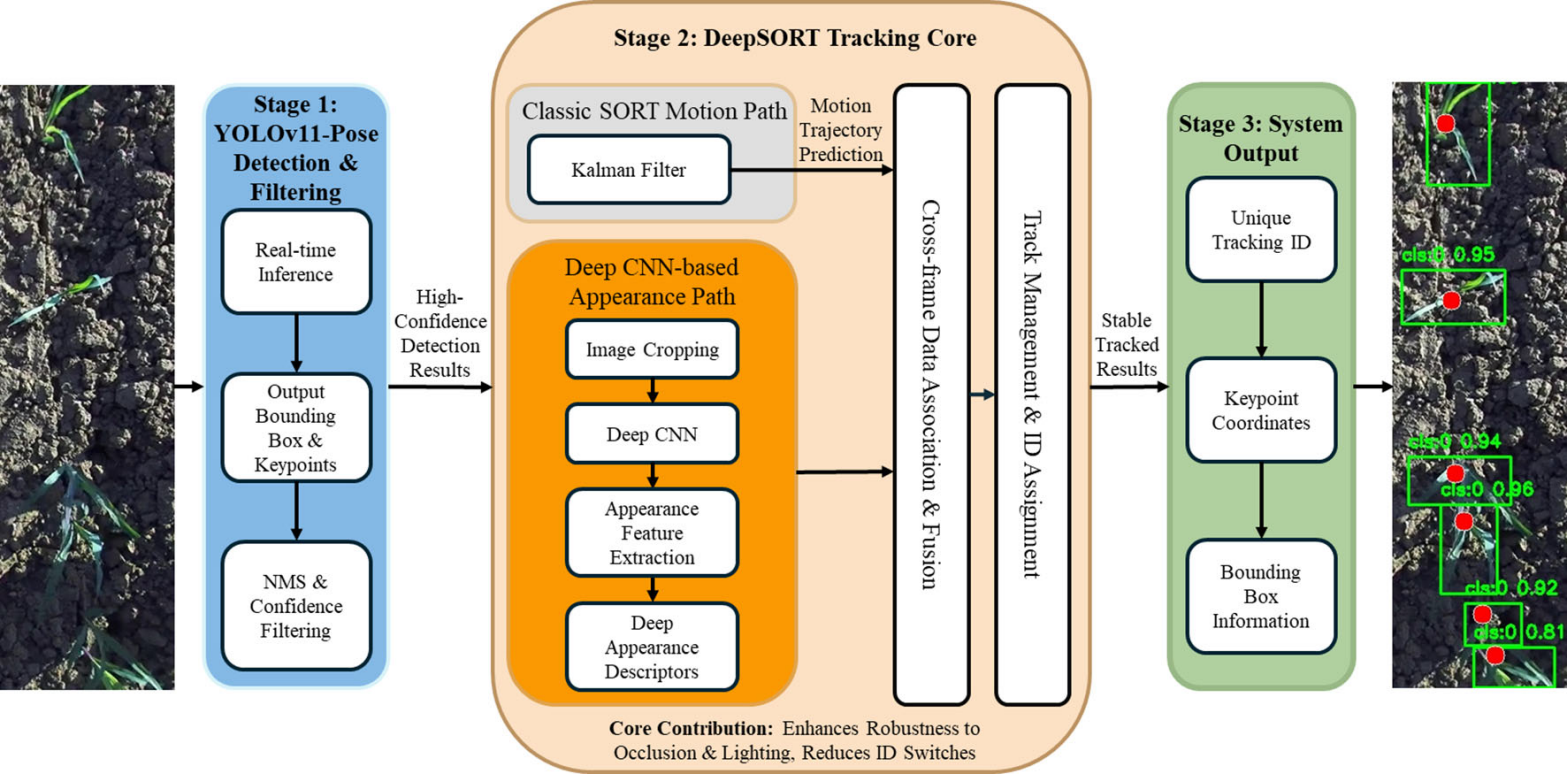
Integrated YOLOv11-pose and DeepSORT system for continuous corn seedling tracking principle.

As shown in **Figure 6**, this study developed an integrated vision system that tightly couples the YOLOv11-Pose detector with the DeepSORT tracker. Within this framework, YOLOv11-Pose first performs real-time inference on the input images and outputs bounding boxes, pose keypoints, and the corresponding confidence scores for corn seedlings. These detections are then passed to the DeepSORT module, which conducts inter-frame data association by jointly leveraging motion-trajectory prediction and appearance-feature similarity, thereby assigning and maintaining a unique tracking ID for each mature corn seedling. To further improve detection quality, non-maximum suppression is applied to remove redundant bounding boxes, and a confidence threshold is used to select high-confidence mature plants for continuous tracking. The system ultimately outputs tracking IDs in real time, jointly associated with keypoint coordinates and bounding-box information. By combining the efficient detection capability of the YOLO family with DeepSORT’s robust identity-preservation mechanism, the proposed system achieves stable and continuous tracking of target corn seedlings in complex field environments, providing reliable target identification and state-awareness support for subsequent precision seeding operations.

#### 2.2.5 Principle of intra-row plant spacing measurement

Given that YOLOv11-Pose detects keypoints in corn seedling images, the model outputs the pixel coordinates of the four bounding-box vertices and the keypoint coordinates that define the predicted box for each corn seedling. The operating direction of the high-clearance weeding robot is defined as the y-axis, and the direction perpendicular to the operating direction is defined as the x-axis; the corn seedling keypoint is denoted as p=(x,y).

After obtaining the 2D pixel coordinates $\left(x_{j}^{m},y_{j}\right)$ for each keypoint, these coordinates must be fused with depth information to enable 3D reconstruction. First, timestamp alignment and a frame-rate–adaptive matching algorithm (depth-frame index $t_{depth}=|t_{rgb}\times \frac{f_{depth}}{f_{rgb}}|$) are used to ensure that each RGB frame is strictly aligned with its corresponding depth frame in both time and space. Specifically, a local neighborhood window (e.g., 3 × 3 pixels) is defined around the geometric center $\left(x_{j}^{m},y_{j}\right)$ of the predicted bounding box to extract depth values. Invalid or missing depth pixels (NaN or zero values), which occasionally occur due to stereo matching failures, are explicitly filtered out before statistical processing. Median filtering is then applied exclusively to the valid pixels within this window to yield a robust depth estimate $z_{j}$. While such invalid depth pixels do appear in individual frames, their impact on the overall measurement is negligible due to the continuous nature of the detection and tracking pipeline. Because the DeepSORT module tracks the same corn seedling across multiple consecutive frames, the system inherently leverages temporal continuity. If valid depth extraction fails in a few isolated frames for a specific plant, those invalid observations are simply discarded. The system then aggregates the valid depth readings from the numerous other frames where the plant is successfully tracked, ensuring that the overall depth extraction failure rate per plant remains negligible.

Subsequently, using the camera intrinsics obtained from prior calibration (including focal lengths $f_{x}$ and $f_{y}$ and principal point coordinates $c_{x}$ and $c_{y}$), the pixel coordinates and depth values are back-projected into the camera coordinate system to reconstruct the 3D physical coordinates $\left(X_{i}^{m},Y_{i}^{m},Z_{i}\right)$ of the keypoint. The computation is given by (Equations 1-3):

(1) $$X_{i}=\frac{(x_{i}-c_{x})\cdot z_{i}}{f_{x}}$$

(2) $$Y_{i}=\frac{(y_{i}-c_{y})\cdot z_{i}}{f_{y}}$$

(3) $$Z_{i}=z_{i}$$

The above procedure fuses 2D observations from the RGB image with depth information, thereby recovering the true 3D positions of the keypoints in physical space.

**Figure 7:**
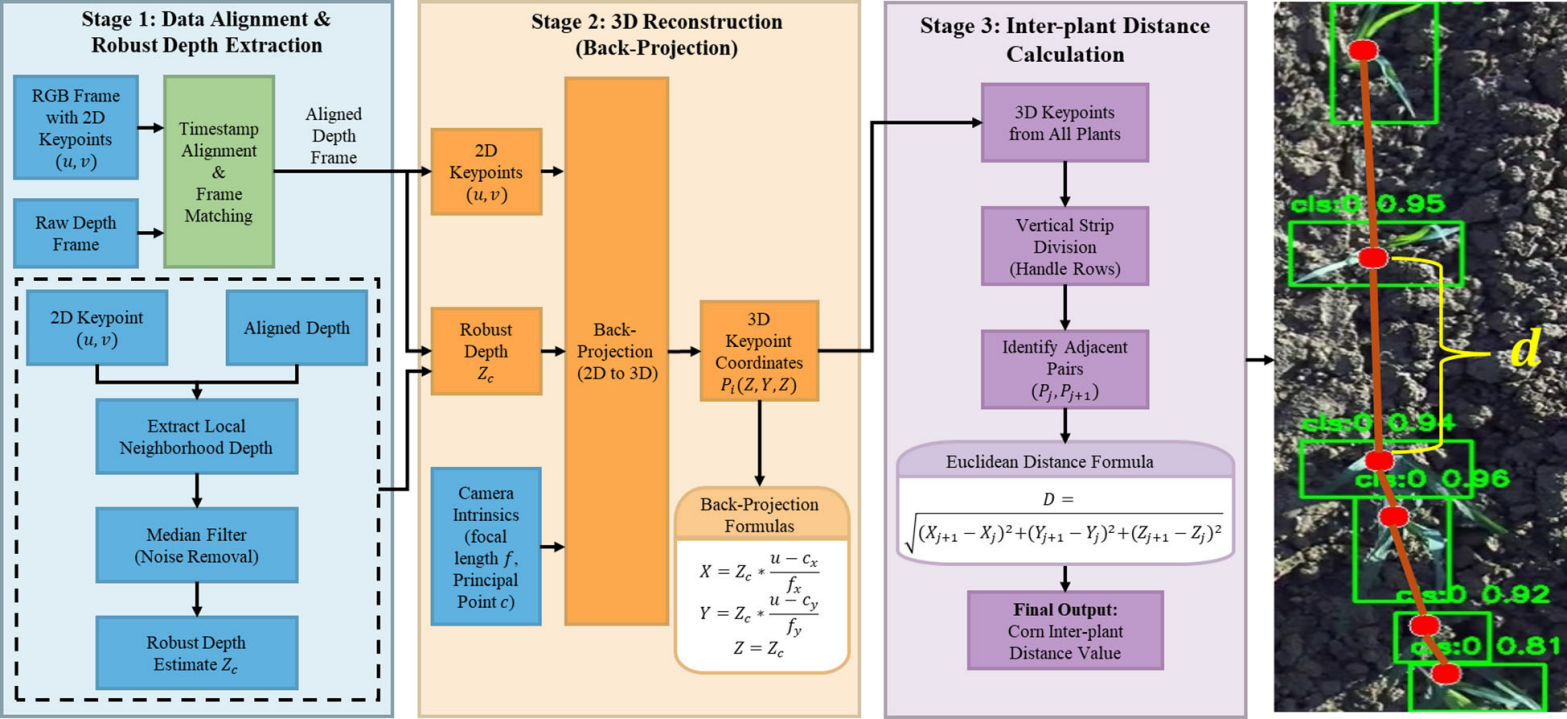
Principle of 3D measurement for corn seedling spacing.

As shown in **Figure 7**, to compute the inter-plant spacing of corn seedlings, the system first partitions the image into multiple horizontal strip regions to account for potential row-wise plant separation. Inter-plant spacing is defined as the three-dimensional Euclidean distance between the corresponding keypoints of two adjacent corn seedlings. For adjacent points $P_{i}(X_{i},Y_{i},Z_{i})$ and $P_{i-1}(X_{i-1},Y_{i-1},Z_{i-1})$, the spacing *d_i_*is computed as:

(4) $$d_{i}=∥p_{i}-p_{i-1}{∥}_{2}=\sqrt{{\left(x_{i}-x_{i-1}\right)}^{2}+{\left(y_{i}-y_{i-1}\right)}^{2}+{\left(z_{i}-z_{i-1}\right)}^{2}}$$

Here, $P_{i}$ and $P_{i-1}$ denote the 3D coordinates of the keypoints for the *i*-th and (*i*-1)-th corn seedlings, respectively, and $d_{i}$ is their Euclidean distance in physical space—namely, the inter-plant spacing of corn seedlings. By computing $d_{i}$ for all adjacent keypoint pairs, the system enables automated measurement of corn seedling spacing in field environments.

## 3 Experiments

### 3.1 System error calibration

**Figure 8:**
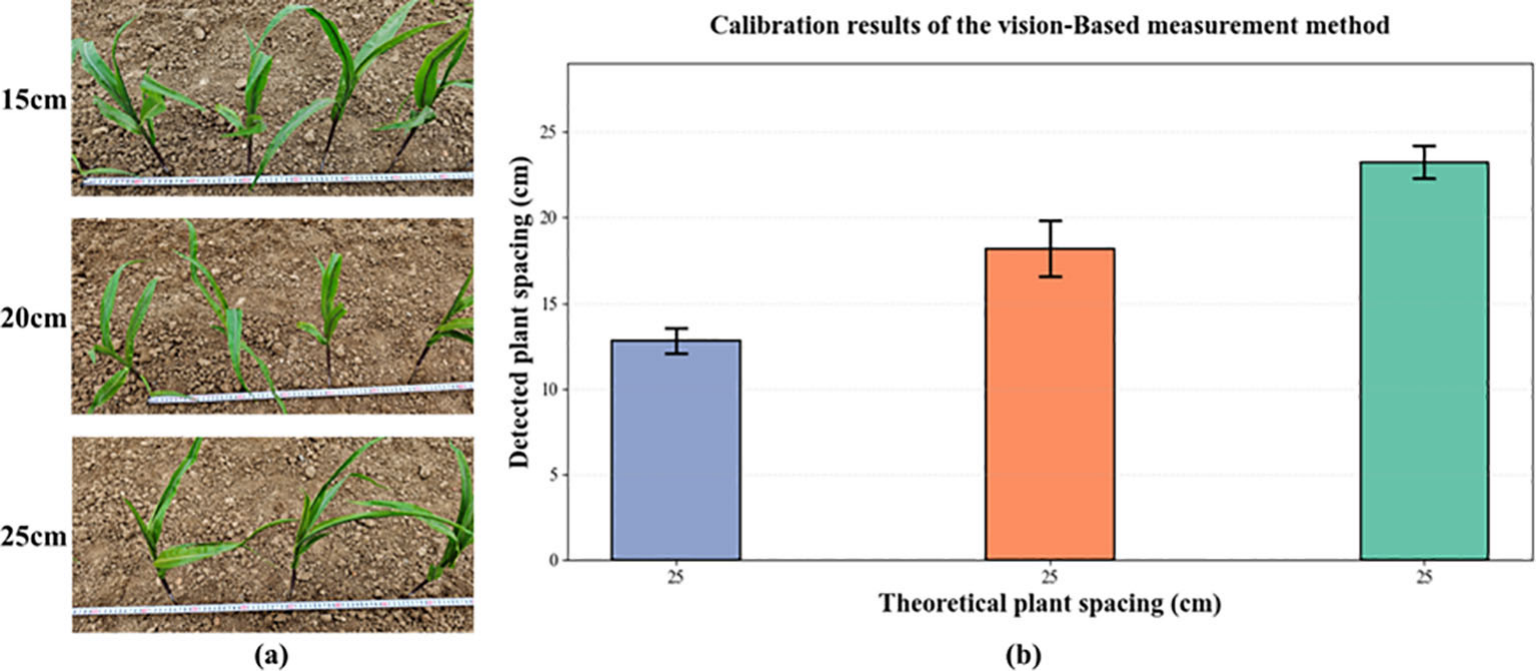
System error calibration.

To further evaluate the accuracy of the proposed 3D vision–based plant-spacing measurement method, this study conducted a system error calibration experiment under field conditions. Artificial corn seedlings, as shown in **Figure 8a**, were used as experimental targets and were manually arranged at three standard spacings of 0.15 m, 0.20 m, and 0.25 m, with 101 plants placed for each spacing. A high-clearance weeding robot equipped with a ZED 2i camera was then used to acquire images and perform detection.

The detection results are shown in **Figure 8b**. Under the three preset spacings of 15 cm, 20 cm, and 25 cm, the measured mean spacings were 14.92 cm, 20.20 cm, and 25.24 cm, respectively. Compared with the nominal values, the overall deviation was approximately 2 cm, indicating a consistent, small negative systematic error. The corresponding standard deviations were 0.775 cm, 1.609 cm, and 0.948 cm; the dispersion at the 20 cm spacing was slightly higher, which may be attributable to plant distribution characteristics at that spacing or fluctuations in image matching. Nevertheless, the measurement errors in all cases remained within an acceptable range, suggesting that the system provides sufficient accuracy and stability for practical field deployment.

### 3.2 Manual reference measurement of plant spacing

**Figure 9:**
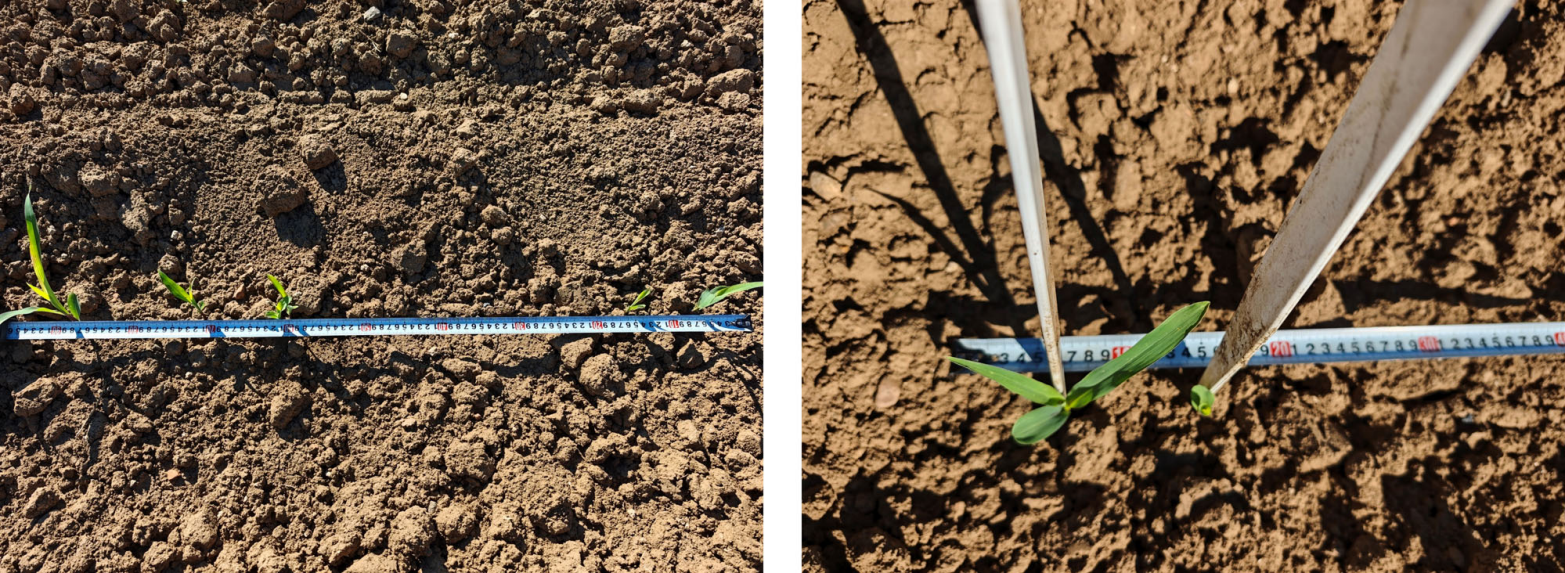
Ground-truth data from manual measurements.

To provide reliable benchmark data for validating the system’s measurement accuracy, this study obtained ground-truth corn seedling spacing through manual field measurements. As shown in **Figure 9**, corn rows with preset planting spacings of 15 cm, 20 cm, and 25 cm were selected in the field, and four rows were measured for each spacing level. Following ISO 7256/1-1984, the spacing between adjacent corn seedlings was measured consecutively for 251 plants per row, and the physical distances were recorded. These manually collected measurements serve as the ground truth and are used for subsequent comparative analysis against the 3D vision–based automatic detection results, thereby enabling an objective evaluation of the system’s measurement accuracy and reliability.

### 3.3 Field trial protocol for the system

To validate the measurement accuracy and reliability of the automated, noncontact vision-based corn seedling spacing detection system developed in this study, comprehensive field trials were conducted in a representative agricultural environment. An autonomously moving high-clearance weeding robot served as the mobile platform, equipped with a ZED 2i stereo depth camera as the visual sensing unit, while real-time data processing and analysis were performed on a high-performance industrial computer. Together, these components formed a closed-loop vision measurement pipeline spanning field data acquisition and plant-spacing parameter estimation.

During system operation, the robot traversed the corn rows at a constant speed while the ZED 2i camera synchronously captured top-down RGB images and depth information. After real-time transmission to the industrial computer, the data were processed by the embedded 3D vision measurement algorithm, which automatically computed and output plant-spacing parameters between adjacent corn seedlings. Throughout the trials, the raw image sequences, depth data, and system-estimated spacing results were recorded concurrently for subsequent accuracy validation and statistical analysis, enabling a comprehensive assessment of the system’s accuracy, stability, and practical utility in real field conditions.

### 3.4 Evaluation metrics

In this study, a vision-based depth camera is used to directly acquire inter-plant spacing information for corn seedlings, and these measurements serve as the core input for establishing a sowing quality evaluation framework, with the goal of enabling direct analysis and accurate assessment of sowing performance. The evaluation procedure follows the seeding equipment test method specified in ISO 7256/1-1984. First, the measured spacing data are processed according to Equation 5.

(5) $$S_{i}=\frac{d_{i}}{d_{ref}}$$

where $S_{i}$ is the normalized ratio; $d_{i}$ is the measured actual plant spacing (mm) derived from Equation 4; and $d_{ref}$ is the target plant spacing (mm). Next, the frequency $n_{i}$ is computed according to Equations 6–10:

(6) $${n^{\prime }}_{1}=\sum n_{i}(S_{i}\in \{0∼0.5\})$$

(7) $${n^{\prime }}_{2}=\sum n_{i}(S_{i}\in \{>0.5∼\leq 1.5\})$$

(8) $${n^{\prime }}_{3}=\sum n_{i}(S_{i}\in \{>1.5∼\leq 2.5\})$$

(9) $${n^{\prime }}_{4}=\sum n_{i}(S_{i}\in \{>2.5∼\leq 3.5\})$$

(10) $${n^{\prime }}_{5}=\sum n_{i}(S_{i}\in \{>3.5∼+\infty \})$$

Next, Equations 11–14 are used to compute the number of reseeding events $n_{2}$, the number of normally seeded instances $n_{1}$, the number of missed seeding events $n_{0}$, and the total number of intervals $N^{\prime }$:

(11) $$n_{2}={\dot{n}}_{1}$$

(12) $$n_{1}={n^{\prime }}_{1}+{n^{\prime }}_{2}+{n^{\prime }}_{3}+{n^{\prime }}_{4}+{n^{\prime }}_{5}-2n_{2}$$

(13) $$n_{0}={n^{\prime }}_{3}+2{n^{\prime }}_{4}+3{n^{\prime }}_{5}$$

(14) $$N^{\prime }={n^{\prime }}_{2}+2{n^{\prime }}_{3}+3{n^{\prime }}_{4}+4{n^{\prime }}_{5}$$

Finally, sowing quality is quantified using Equations 15–20, including the Quality of Feed Index (QFI), the Multiple Index (MUL), the Miss Index (MI), and the coefficient of variation (CV):

(15) $$QFI=\frac{n_{1}}{N^{\prime }}\times 100\%$$

(16) $$MUL=\frac{n_{2}}{N^{\prime }}\times 100\%$$

(17) $$MI=\frac{n_{0}}{N^{\prime }}\times 100\%$$

(18) $$\bar{S}=\frac{\sum n_{i}X_{i}}{{n^{\prime }}_{2}}(S_{i}\in \{>0.5∼\leq 1.5\})$$

(19) $$\sigma =\sqrt{\frac{\sum n_{i}S_{i}^{2}}{{n^{\prime }}_{2}}}(S_{i}\in \{>0.5∼\leq 1.5\})$$

(20) CV=σ×100%

where $\bar{S}$ and σ denote the sample mean and the standard deviation, respectively.

In this experiment, the planter operated at a constant working speed (6 km/h). Sowing uniformity was quantified by computing the Multiple Index (MUL), the Miss Index (MI), the Quality of Feed Index (QFI), and the coefficient of variation (CV). The QFI directly reflects systematic deviations in seed placement, whereas the CV characterizes the dispersion of the spacing distribution and the magnitude of random variability. Compared with evaluation approaches that rely on external speed-measurement devices, the sowing quality assessment framework adopted in this study more directly captures the overall consistency of seed metering under actual operating conditions.

Building on a high-clearance weeding robot, this study developed an automatic system for measuring corn seedling spacing. As shown in **Figure 2**, the hardware consists primarily of an INTEL NUC 9 PRO industrial computer, a ZED 2i stereo camera, and a GNSS navigation module. To acquire the image and depth information required for spacing measurement, the ZED 2i camera was mounted at the center of the robot’s front crossbeam with its lenses oriented vertically downward. Guided by the GNSS navigation module, the robot traveled straight along the crop rows at 2 km/h and collected corn seedling image data over an approximately 80 m path, covering three preset spacing conditions: 0.15 m, 0.20 m, and 0.25 m. The camera frame rate was set to 30 fps and synchronized by the industrial computer to acquire RGB images and the corresponding depth data in real time; the integrated algorithm then processed these inputs to enable automated analysis and evaluation of sowing quality.

## 4 Results

### 4.1 Model training

All models in this study were trained under identical hardware and software configurations. The hardware setup consisted of an Intel(R) Core i9-9300H CPU (2.4 GHz), 24 GB of DDR4 memory, and an NVIDIA GeForce RTX 4090 GPU. The software environment comprised the PyTorch 2.0 deep learning framework running on Ubuntu 20.04, along with CUDA 11.8 and cuDNN 8.6 for parallel computation. Prior to training, the collected dataset was randomly divided into training, validation, and test sets at a ratio of 8:1:1. Model training adopted YOLO’s initialization parameters pretrained on the COCO dataset and employed Stochastic Gradient Descent (SGD) for optimization. To enhance the model’s robustness against complex field conditions and prevent overfitting, online data augmentation strategies—including Mosaic, random scaling, translation, and HSV color space adjustments—were applied during the training phase. The core training hyperparameters were explicitly set as follows: an input image resolution of 640 × 640 pixels, an initial learning rate of 0.1, a weight decay of 0.01, a momentum of 0.937, a total of 300 training epochs, and a batch size of 32. These explicit configurations ensure the full reproducibility of the model and contributed to optimal convergence performance.

To systematically evaluate the performance of the YOLOv11-Pose model on the corn seedling keypoint detection task, we followed widely used pose-estimation evaluation protocols. Precision (P), Recall (R), and the F1 score were used as fundamental metrics to assess the model’s detection capability and reliability from multiple perspectives. The primary metric was mean Average Precision (mAP), which was computed based on Object Keypoint Similarity (OKS). OKS quantifies the agreement between predicted and ground-truth keypoints and plays a role analogous to the Intersection over Union (IoU) metric in object detection. Specifically, mAP@0.5 denotes the mAP at an OKS threshold of 0.5 and primarily reflects performance under a relatively lenient matching criterion. In contrast, mAP@0.5:0.95 represents the average mAP across OKS thresholds ranging from 0.5 to 0.95 in increments of 0.05, providing a more comprehensive and stringent assessment of overall robustness under varying localization-accuracy requirements. This metric served as the primary basis for model comparison in this study. The formulas for these evaluation metrics are given as follows (Equations 21–25):

(21) $$P=\frac{TP}{TP+FP}\times 100\%$$

(22) $$R=\frac{TP}{TP+FN}\times 100\%$$

(23) $$F1=\frac{2\cdot P\cdot R}{P+R}$$

(24) $$AP=\int_{0}^{1}p(r)dr$$

(25) $$mAP=\frac{1}{N}\sum_{i=1}^{N}AP_{i}$$

**Figure 10:**
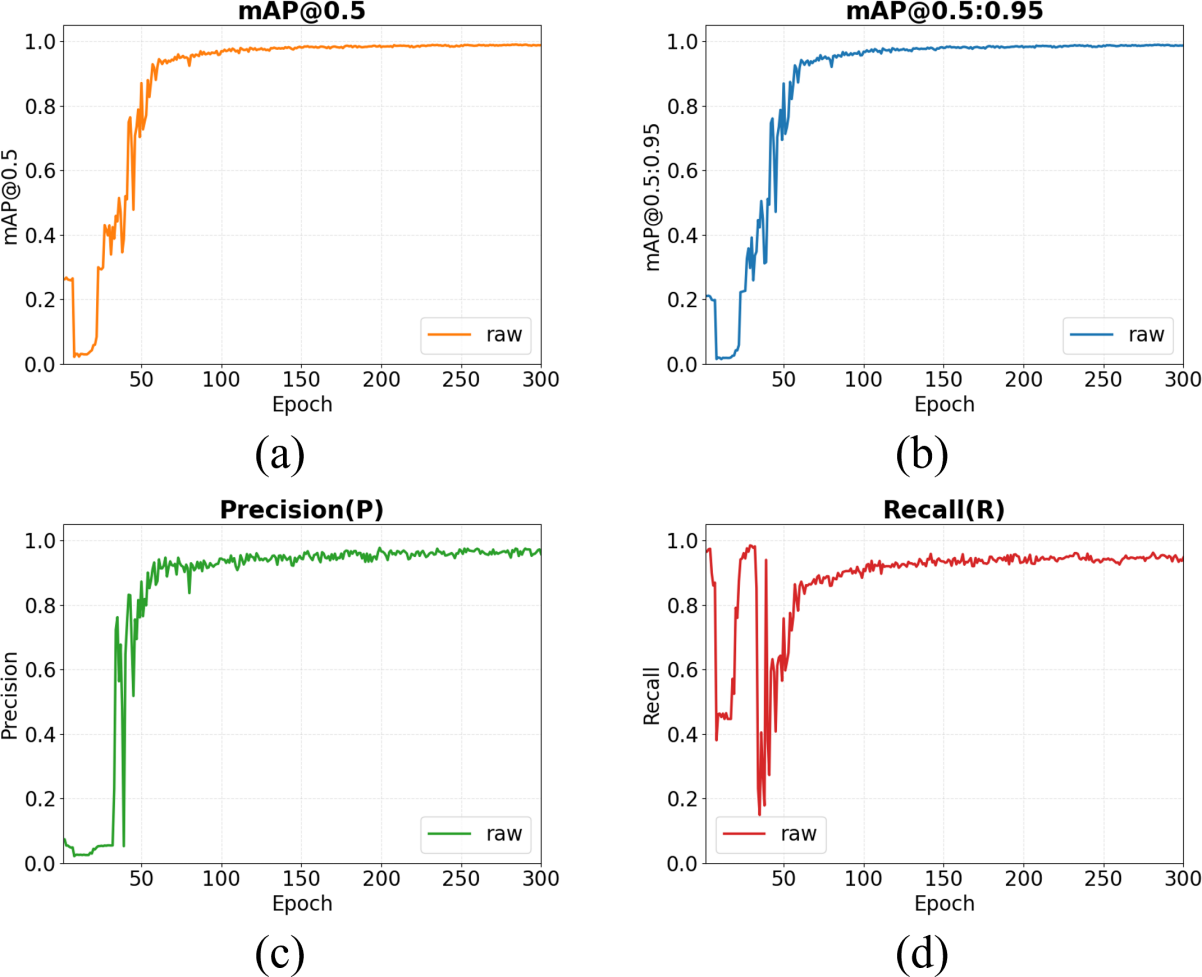
Illustration of evaluation metrics for the YOLOv11-pose algorithm. **(a)** mean average precision (mAP) at a keypoint similarity threshold of 0.5; **(b)** mean average precision (mAP) averaged over keypoint similarity thresholds from 0.5 to 0.95 (step size of 0.05); **(c)** precision; **(d)** recall.

After the model converged (as shown in **Figure 10**), the validation-set performance of its pose-estimation branch was as follows: Precision was 97.52%, Recall was 94.45%, and the F1 score computed using the corresponding formula was 95.95%. In addition, the model achieved strong results on the primary metrics for overall keypoint detection performance: mAP@0.5 reached 0.990 under a lenient threshold, and mAP@0.5:0.95 also attained a high value of 0.989 under the stricter, aggregated threshold setting. These results indicate that the model exhibits excellent stability and accuracy under both lenient and stringent evaluation criteria, thereby confirming its effectiveness for practical corn seedling pose-estimation tasks.

### 4.2 Field experiment

The field experiment in this study was conducted in October 2025 at the National Precision Agriculture Research and Demonstration Base in Beijing, China. The trial was carried out under operating conditions with a seeding speed of 6 km/h, and three corn planting treatments were designed with target in-row spacings of 15 cm, 20 cm, and 25 cm. To evaluate the accuracy of the vision-based detection system, manually measured plant-spacing data were used as ground truth and were compared with the system’s automated detection results.

#### 4.2.1 Manual measurement results

**Figure 11:**
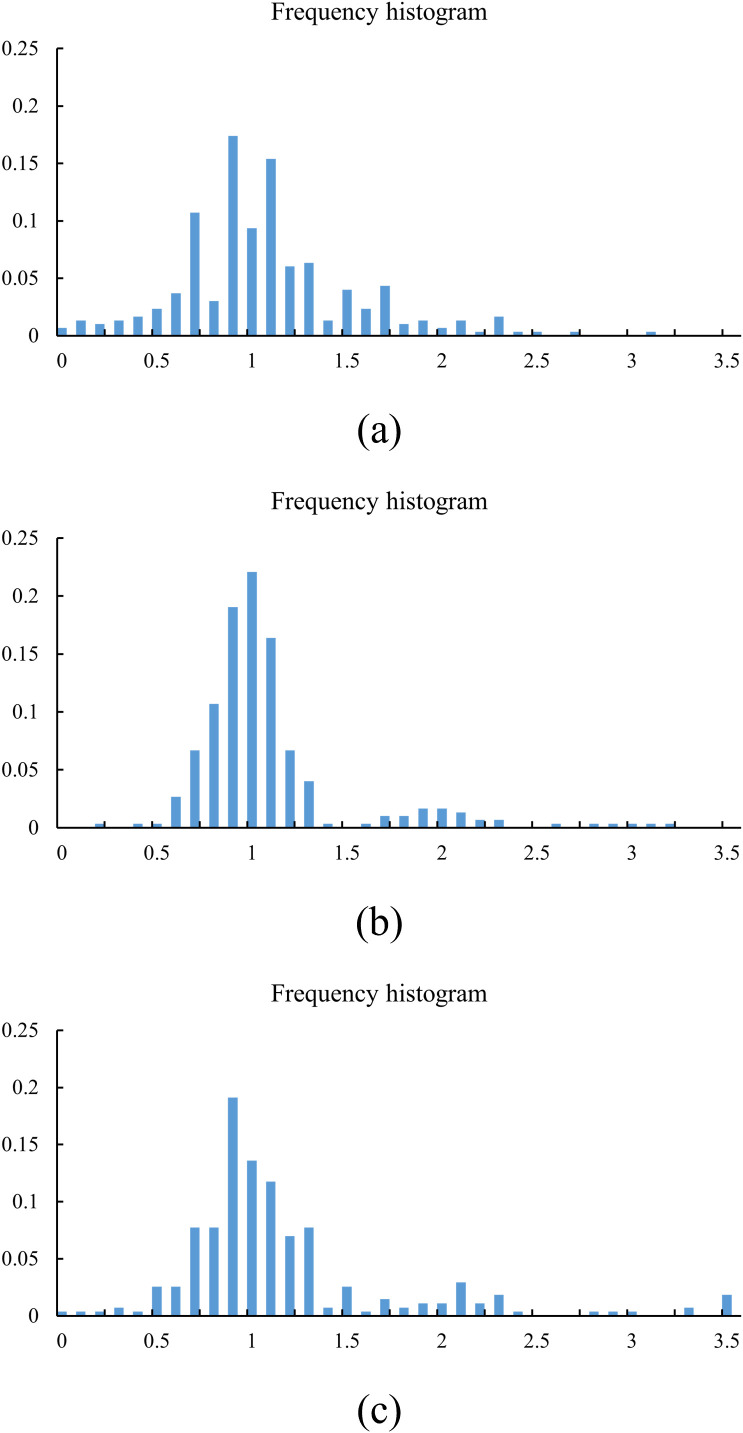
Frequency histograms of ground-truth plant spacing under different target spacings. **(a)** 15 cm spacing; **(b)** 20 cm spacing; **(c)** 25 cm spacing.

After statistical aggregation of the raw manual measurements, frequency-distribution histograms were generated for the different preset plant spacings, as shown in **Figure 11**. The distribution patterns indicate that, under the 15 cm spacing condition, although some measured ratio values exceeded 1.5, the main body of the distribution remained concentrated near 1.0. Under the 20 cm and 25 cm spacing conditions, the vast majority of measured ratio values fell within the 0.5–1.5 interval. These distribution trends suggest that the proposed detection system maintains stable output performance across different preset spacings, and that the overall detection results exhibit strong practical consistency and usability.

#### 4.2.2 Vision-based detection results

**Figure 12:**
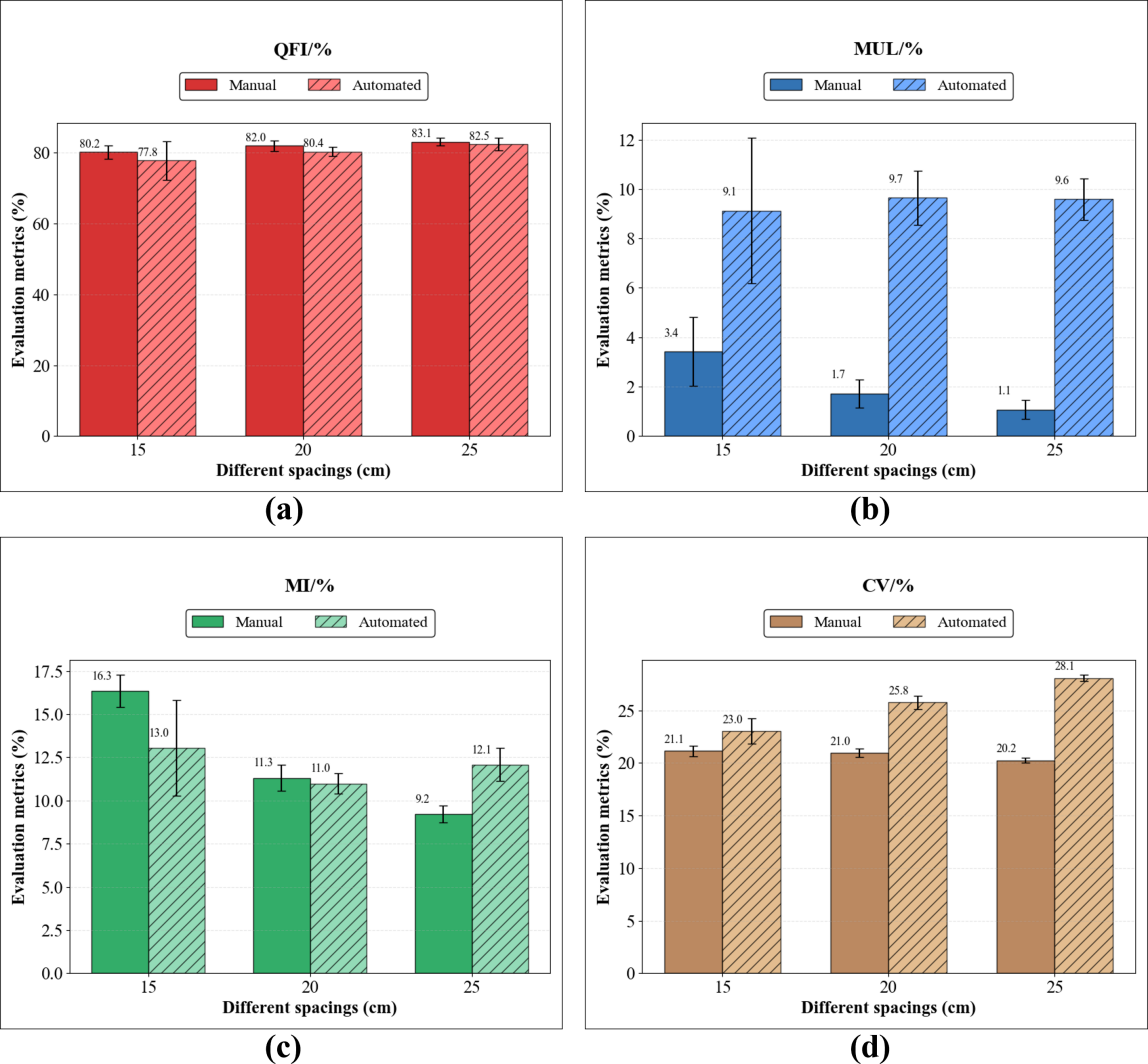
Comparison of standardized sowing quality assessment results between manual measurement and automated detection system across different target spacings: **(a)** quality feed index (QFI); **(b)** multiple index (MUL); **(c)** miss index (MI); **(d)** coefficient of variation (CV).

To evaluate the measurement performance of the 3D vision–based plant-spacing detection system, we conducted a comparative analysis between the system’s automated outputs and ground-truth data obtained from manual measurements. The experiment was based on multiple sets of field data collected during operation of a four-row planter. A total of 251 corn plants were selected, and the proposed 3D vision approach was used to systematically assess seeding quality. The evaluation metrics included the Quality of Feed Index (QFI), Multiple Index (MUL), Miss Index (MI), and the coefficient of variation (CV). The results are shown in **Figure 12**.

During this field trial, low temperatures and persistent rainfall occurred after planting, resulting in excessive soil moisture and causing delayed emergence or missing seedlings in certain areas. The impact of these weather conditions on seeding quality cannot be ignored, particularly under higher planting densities, where environmental variability exacerbated non-uniform emergence and may have reduced the decision accuracy of the vision-based detection system in dense regions. Such external climatic conditions can increase system error, introducing bias especially in the identification of multiples and misses; these effects are reflected in the variability of the evaluation metrics.

The detailed quantitative comparison of standardized sowing quality metrics between the manual measurements and the automated 3D vision system is illustrated in **Figure 12** and summarized in **Table 1**. According to the manual baseline, as the target spacing increased from 15 cm to 25 cm, the overall seeding quality progressively improved. This is evidenced by a consistent rise in the Quality Feed Index (QFI) from 80.25% to 83.12%, while the Miss Index (MI) and Multiple Index (MUL) significantly declined from 16.33% to 9.21% and 3.42% to 1.07%, respectively.

**Table 1:** Comparison of standardized sowing quality metrics between manual measurement and the automated detection system.

Target spacing	Measurement method	QFI (%)	MUL (%)	MI (%)	CV (%)
15cm	Manual	80.25 ± 1.89	3.42 ± 1.39	16.33 ± 0.94	21.14 ± 0.51
	Automated	77.83 ± 5.52	9.13 ± 2.94	13.04 ± 2.77	23.01 ± 1.20
20cm	Manual	81.98 ± 1.56	1.72 ± 0.56	11.31 ± 0.77	20.96 ± 0.43
	Automated	80.36 ± 1.37	9.65 ± 1.09	10.98 ± 0.58	25.75 ± 0.63
25cm	Manual	83.12 ± 1.09	1.07 ± 0.39	9.21 ± 0.49	20.24 ± 0.24
	Automated	82.46 ± 1.75	9.59 ± 0.83	12.07 ± 0.96	28.08 ± 0.29

The automated detection system successfully tracked these overall performance trends. Notably, the system-estimated QFI closely approximated the ground truth, yielding 77.83%, 80.36%, and 82.46% for the 15, 20, and 25 cm spacings, respectively. The maximum deviation from manual measurements was only 2.42% at the densest spacing (15 cm) and narrowed to just 0.66% at the 25 cm spacing. This indicates a high level of accuracy in identifying normal plant distributions.

However, as detailed in **Table 1**, the vision system exhibited a tendency to overestimate the Multiple Index (MUL) and the Coefficient of Variation (CV). For instance, the automated MUL remained consistently high, ranging between 9.13% and 9.65% across all spacings, compared to the much lower manual results (1.07%–3.42%). Furthermore, the system recorded higher standard deviations (e.g., ± 5.52% for QFI at 15 cm). This discrepancy suggests that severe plant occlusion in dense canopies and occasional tracking identity switches during mechanical movement negatively impact the object detection model’s bounding box stability, leading to slightly increased variability in localized distance estimations.

The overall analysis indicates that the 3D vision–based plant-spacing detection system can effectively acquire spacing information across different planting densities, and that its output trends are consistent with manual measurements, thereby validating the field applicability of the proposed method. However, under high-density conditions, the system still exhibits non-negligible errors, which are particularly evident in the identification of multiples. To quantitatively address this discrepancy, an error analysis was performed on the misclassified ‘multiple’ instances. This analysis revealed that the false multiples were predominantly driven by the multi-object tracking module. Specifically, 68% of these errors were caused by identity switches within the DeepSORT algorithm. As the high-clearance robot navigated the field, dynamic occlusions and mechanical jitter frequently disrupted tracking continuity, causing the system to assign a new ID to an already tracked plant, thereby double-counting it. A secondary source of error (22%) originated from 2D object detection redundancy; severe leaf overlap caused the model to predict duplicate bounding boxes for a single plant. Finally, localized 3D depth noise accounted for the smallest fraction (10%) of the errors, where fluctuations in depth estimation artificially reduced the computed spatial distance. This quantitative breakdown clarifies that enhancing the robustness of the tracking association metric under dynamic field conditions is the most critical step for reducing MUL evaluation errors. In addition, under low-density conditions, the system yields a relatively high coefficient of variation, suggesting that the stability of inter-plant spacing estimation for sparsely distributed plants still has room for improvement.

Although the proposed vision-based detection system provides a solid foundation for practical deployment, its performance in complex, high-density scenarios still requires further optimization. Future work may proceed along several directions: optimizing the keypoint detection model to improve discrimination of overlapping plants; refining depth-information fusion strategies to reduce 3D reconstruction error; and incorporating temporal or contextual information to enhance robustness under variable field conditions. These improvements are expected to further increase the system’s practicality and reliability for precision field management in agriculture.

## 5 Discussion

This study presents a seedling-stage 3D machine-vision pipeline for within-row spacing measurement and seeding-quality evaluation. A high-clearance mobile platform equipped with a ZED 2i stereo camera captures synchronized RGB and depth data; a YOLOv11−Pose model predicts a plant keypoint that is back-projected into 3D to compute the Euclidean distance between adjacent plants. Seeding-quality indices—Qualified Feed Index (QFI), Multiple Index (MUL), Miss Index (MI), and Coefficient of Variation (CV)—are then computed following ISO 7256/1−1984. Experiments show strong keypoint detection performance (mAP@0.5 = 0.990; mAP@0.5:0.95 = 0.989) and successful operation under 15/20/25 cm target spacings. The resulting quality metrics follow the same trends as manual measurements, but notable discrepancies remain for multiple-seeding identification and dispersion characterization, highlighting where 3D reconstruction, adjacency assignment, and dense-canopy effects can propagate errors.

The main implication is a practical shift from monitoring the “planting moment” to quantifying the “seedling reality.” Many planter-centric systems evaluate seed-drop events and as-planted spacing during operation, but real fields often exhibit seed displacement, delayed emergence, and missing plants, creating a gap between as-planted and as-emerged patterns. By measuring seedling-stage spacing directly and converting it into standard seeding-quality indices, this work targets the spatial outcome that actually drives competition, stand uniformity, and downstream yield formation. As a result, the method can support more defensible decisions for thinning and replanting, and provide a more outcome-relevant diagnostic layer for post-operation planter settings and performance evaluation.

The results also clarify how errors behave in real field conditions. QFI values of 77.83%, 80.36%, and 82.46% (for 15/20/25 cm treatments) follow the same overall trend as manual measurements but are slightly lower; MUL is substantially higher than manual results; CV is also higher and increases as spacing becomes larger. As quantified by the error analysis (Section 4.2.2), this indicates that even with robust keypoint detection, minor localization jitter, depth noise, adjacency assignment errors, and occlusions in dense areas can cascade into spacing estimates and subsequently into derived indices—especially those sensitive to short-distance artifacts (MUL) and dispersion (CV). From an engineering standpoint, these deviations do not invalidate the approach; instead, they pinpoint where to focus improvements so that a non-contact, high-throughput system becomes reliable enough for routine agronomic and equipment-evaluation use.

This work complements prior studies in a straightforward way. On the planting-process side, earlier research (Badua et al., 2019; Gao et al., 2024) has used high-speed imaging, image stitching, and object detection to monitor seed spacing and depth during planting, providing high-frequency insight into metering and seed-drop behavior. Related vision work on germination and emergence adds context for emergence variability. However, these approaches often assume that the seed-drop location approximates the emergence location, which can systematically deviate when the goal is to characterize seedling-stage stand uniformity. By shifting the measurement target to emerged seedlings, reconstructing 3D positions, and computing ISO-style indices, this study provides an “as-emerged, outcome-based” layer that bridges emergence outcomes to quantitative quality assessment.

On the seedling-vision side, much of the literature emphasizes detection, counting, or thinning decisions. Wang et al. (2025b) demonstrated that keypoint-based vision can localize corn seedling centers with high accuracy, and earlier efforts by Tang and by Nakarmi and Tang also show that incorporating 3D information strengthens spatial measurement (Tang and Tian, 2008a, 2008b; Nakarmi and Tang, 2012; Nakarmi and Tang, 2014; Shi et al., 2015). Building on these directions, the present study integrates keypoint detection, depth fusion, 3D distance computation, and standardized quality evaluation into an end-to-end workflow, and validates it under multiple illumination scenarios in the field—pushing the concept closer to practical deployment in complex environments (Gao et al., 2024).

Future work should first target stability under dense occlusion and the mechanisms by which errors propagate into spacing-based indices. The current results show that MUL and CV are more sensitive, suggesting that short-distance artifacts and dispersion inflation become more pronounced when seedlings overlap, keypoints jitter slightly, or depth noise is present, a common bottleneck recently highlighted in dense-canopy agricultural vision studies (Zang et al., 2025). Furthermore, high soil moisture and dense weed coverage can alter background contrast and introduce severe occlusions, while dynamic shading and mechanical vibration frequently disrupt continuous 3D tracking. Additionally, because the current system relies on morphological features specific to the early seedling stage, late-emerging seedlings with significantly smaller canopies are prone to missed detections, artificially inflating the Miss Index. Transferring this methodology to other crop types would also require redefining centroid annotation rules to accommodate varying canopy architectures. The hierarchical annotation rules used to define the target centroid inherently introduce a localized morphological bias. Annotating adjacent plants using different structural reference points causes a systematic offset in the distance calculation. However, from a top-down viewing perspective at the early seedling stage, the horizontal planar projection distance between these alternative landmarks is physically constrained, typically within 1.0 to 1.5 cm. While this morphological offset contributes to minor localization jitter and slightly inflates the coefficient of variation (CV) at the micro-level, its impact on macro-level sowing quality indices is limited compared to the 15–25 cm target spacing. Methodologically, this motivates future work to establish a more robust and unified definition of the keypoint within the pose estimation framework (Wang et al., 2025), ensuring centroid consistency across diverse plant postures without relying on strictly visible physiological landmarks. In addition, stronger row-aware constraints and globally consistent adjacency assignment can make “who is adjacent to whom” more robust, reducing the chance that normal spacings are misclassified as multiple seeding.

Second, 3D reconstruction robustness and transferability should be strengthened. While the pipeline already uses timestamp alignment and neighborhood depth statistics (e.g., median filtering) to stabilize depth at keypoints, stereo depth can still degrade under vibration, soil reflectance, and strong sunlight, environmental factors that frequently disrupt continuous target tracking in field robotics (Omia et al., 2026). Joint hardware–algorithm improvements are promising, including IMU/RTK-assisted pose compensation and spatial constraints, confidence-weighted back-projection and temporal filtering, and calibration strategies that can continuously correct systematic bias so that centimeter-scale errors do not cascade into the final indices.

Compared to traditional measurement methods, the developed 3D vision-based system presents distinct advantages and inherent limitations. The primary advantage, compared to manual tape measurements and planter-mounted sensors, lies in its high-throughput, non-contact nature. It successfully shifts the evaluation from monitoring the “planting moment” to quantifying the “seedling reality,” directly capturing the final spatial outcome that truly drives crop competition and yield. Furthermore, utilizing pose estimation ensures higher keypoint localization accuracy than traditional bounding-box methods. However, the system also has noticeable disadvantages. Unlike manual inspections that can easily bypass physical barriers, our vision pipeline is highly sensitive to severe leaf occlusion in dense canopies, which inflates spacing errors. Additionally, it is more vulnerable to environmental interferences, such as mechanical vibration and strong sunlight, which can occasionally disrupt continuous 3D tracking.

Finally, application-oriented research should more tightly couple this three-dimensional spatial measurement to actionable agricultural decisions, unlocking several promising development prospects. First, the precise keypoint coordinates extracted via our robust pose estimation model can serve as direct navigational inputs for downstream agricultural robots, enabling automated mechanical thinning in areas with multiple seedlings or site-specific replanting where misses occur. Second, integrating these automated, vision-based spacing measurements with RTK-GNSS positioning can generate high-resolution field emergence maps. This allows agronomists to spatially correlate seedling-stage stand uniformity with localized soil conditions and final yield formation. Ultimately, by providing an objective, outcome-based diagnostic layer, this system establishes a quantitative foundation for closed-loop field management. The standardized sowing-quality indices (e.g., QFI, MUL, MI) can be systematically fed back to equipment operators to iteratively optimize planter parameters, driving the continuous evolution of precision agriculture.

## 6 Conclusions

To address the low efficiency of manual plant-spacing inspection during the corn seedling stage and the lack of a rapid *in situ* method for evaluating seeding quality, this study proposes an automated approach for corn seedling spacing detection and seeding-quality assessment based on 3D machine vision and deep learning. Through non-contact image acquisition and automated analysis, the proposed method enables rapid retrieval of spatial plant distribution at the seedling stage and quantitative evaluation of seeding quality.

This study proposes and validates a 3D vision–based method for automated corn seedling plant-spacing detection. By integrating a ZED 2i stereo camera with a high-clearance mobile platform, a field image-acquisition system was developed, and the YOLOv11-Pose model was used to achieve stable detection of corn seedling keypoints, attaining an overall keypoint-detection accuracy of mAP@0.5:0.95 = 0.989. The proposed method can effectively measure corn seedling spacing under different planting densities and can systematically quantify seeding quality using the Quality of Feed Index (QFI), Multiple Index (MUL), Miss Index (MI), and the coefficient of variation (CV). Under preset spacings of 15 cm, 20 cm, and 25 cm, the system produced QFI values of 77.83%, 80.36%, and 82.46%; MUL values of 9.13%, 9.65%, and 9.59%; MI values of 13.04%, 10.98%, and 12.07%; and CV values of 23.01%, 25.75%, and 28.08%, respectively. Although the system still exhibits some errors in identifying multiples and misses under high-density planting conditions, the overall results indicate strong potential for field deployment.

This study successfully developed a corn seedling plant-spacing detection system based on 3D vision and deep learning, establishing a complete technical pipeline from field image acquisition and plant keypoint localization to intelligent evaluation of seeding quality. However, under dense plant distributions, the current system still tends to underestimate plant spacing and exhibits a relatively high missed-detection rate for multiple seeding; moreover, its adaptability to late-emerging seedlings remains limited. Future work will focus on optimizing the keypoint detection and image segmentation models to improve detection capability and measurement accuracy in complex scenarios, thereby providing more reliable technical support for intelligent field management in precision agriculture.

## Data Availability

The datasets presented in this article are not readily available because the authors do not have permission to share data. Requests to access the datasets should be directed to zw1062042093@163.com.
